# The value of EQ-5D-3L and EQ VAS as a patient-reported outcome measure for patients with ankylosing spondylitis in routine healthcare: an evaluation of construct validity and responsiveness based on the Swedish Rheumatology Quality Register

**DOI:** 10.1186/s41687-026-01009-0

**Published:** 2026-02-07

**Authors:** Kinza Degerlund-Maldi, Malin Regardt, Camilla Nystrand Länsman, Lena Larsson, Ioannis Parodis, Emelie Heintz

**Affiliations:** 1 Health Economic and Policy Research Group, Department of Learning, Informatics, Management and Ethics, Karolinska Institutet, Stockholm, Sweden; 2 Stockholm Center for Health Economics, Center for Health Economics, Informatics and Health Services Research (CHIS), Stockholm Health Care Services, Stockholm, Sweden; 3 Medical Unit Allied Health Professionals, Karolinska University Hospital, Stockholm, Sweden; 4 Division of Occupational Therapy, Department of Neurobiology, Care Sciences and Society, Karolinska Institutet, Stockholm, Sweden; 5 Institute of Odontology, Sahlgrenska Academy, University of Gothenburg, Gothenburg, Sweden; 6 Division of Rheumatology, Department of Medicine Solna, Karolinska Institutet, Karolinska University Hospital, and Center for Molecular Medicine (CMM), Stockholm, Sweden; 7 Department of Rheumatology, Faculty of Medicine and Health, Örebro University, Örebro, Sweden

**Keywords:** Health economics, Patient-reported outcome measures, Quality of life, Psychometric properties, Rheumatology, EQ-5D-3L, EQ VAS

## Abstract

**Background:**

EQ-5D-3L is a generic health-related quality of life (HRQoL) instrument, widely used in both economic and non-economic research. The purpose of this study is to examine the construct validity and responsiveness of EQ-5D-3L (descriptive system and EQ-5D-3L index), as well as EQ VAS in patients with ankylosing spondylitis (AS).

**Methodology:**

The study is based on real-world individual-level data from the Swedish Rheumatology Quality Register. To assess construct validity and responsiveness, we formulated and tested hypotheses concerning expected correlations between EQ-5D-3L and EQ VAS scores and those of comparator instruments (Bath Ankylosing Spondylitis Disease Activity Index, Ankylosing Spondylitis Disease Activity Score, Bath Ankylosing Spondylitis Functional Index, visual analogue scale for pain, fatigue, and general health), as well as expected differences between patient groups stratified by disease activity or physical function. Further, the area under the receiver operating characteristic curve was analysed. At least 75% of the predetermined hypotheses had to be confirmed to support construct validity or responsiveness.

**Results:**

In total, 4,878 patients with AS were included in this study. As expected, the correlations between EQ-5D-3L and EQ VAS scores and those from the comparator instruments were moderate or strong. Further, EQ-5D-3L and EQ VAS were able to detect expected differences between groups. Changes in EQ VAS over time also showed moderate to strong correlations with corresponding changes in comparator instruments. However, the changes in the EQ-5D-3L dimensions self-care and usual activities had weaker correlations than expected with changes in the comparator instruments.

**Conclusion:**

Over 75% of the hypotheses for construct validity of EQ-5D-3L and EQ VAS were confirmed, supporting their construct validity in patients with AS. Responsiveness of EQ VAS was also supported. However, limited responsiveness of the dimensions in the EQ-5D-3L indicates that the descriptive system and the EQ-5D-3L index may not fully capture changes over time among patients with AS.

**Supplementary Information:**

The online version contains supplementary material available at 10.1186/s41687-026-01009-0.

## Background

Ankylosing spondylitis (AS) is an inflammatory rheumatic disease including symptoms such as back pain, stiffness, and fatigue [1], leading to impaired health-related quality of life (HRQoL) [2]. In this patient group, the generic HRQoL instrument EQ-5D has been used to evaluate the effect of medications on HRQoL [3, 4] and in economic evaluations of AS treatments [5]. Originally developed for estimating the value of health for health economic evaluations [6–8], EQ-5D consists of a descriptive system with five items (health dimensions), which can be summarised into an index value representing the overall value of the health state of the patient, and a visual analogue scale (EQ VAS). It is one of the most commonly used generic preference-based instruments, and it is extensively applied in both economic and non-economic research [9–11].

Over time, EQ-5D has been adopted in routine healthcare settings to monitor treatment outcomes and support clinical decision-making [12, 13], and assessing HRQoL has been recognized as a crucial component in the management of AS, including therapeutic decision-making [14]. In Sweden, patients with AS complete the three-level version of EQ-5D (EQ-5D-3L) and the EQ VAS in conjunction with their follow-up visits, and the data can support shared decision-making between patients and healthcare providers [12]. In such contexts, it is recommended that emphasis be placed on the five dimensions in the descriptive system of the instrument [15]. The descriptive system can provide a more nuanced picture of the patient’s health than the overall index value [16].

A key consideration when choosing between instruments is whether they demonstrate adequate psychometric properties for the intended purpose and target population [17]. An instrument should accurately measure the concept it is intended to measure, a property known as construct validity, and be able to capture changes over time, a property known as responsiveness [17]. Thus, if the EQ-5D-3L is to be used to measure HRQoL in patients with AS, the results should be an accurate reflection of the patients’ HRQoL. If an instrument exhibits poor construct validity or poor responsiveness, it may fail to accurately reflect patients’ HRQoL or changes therein, thereby potentially providing inaccurate information about them.

As measurement properties can vary depending on the population studied, construct validity and responsiveness should be assessed across different populations [18]. Three previous studies support the construct validity of both the EQ-5D-3L index and the EQ VAS in patients with AS [19–21], and one study also supports the responsiveness of the EQ VAS [20]. However, the support for the responsiveness of the EQ-5D-3L index is inconsistent [20, 21]. Regarding the descriptive system, one study (*n* = 90) found that patients with AS who reported problems with the EQ-5D-3L dimensions mobility, self-care, and usual activities had statistically significantly higher scores on the disease-specific physical function instrument Bath Ankylosing Spondylitis Functional Index (BASFI) than those reporting no problems [19]. Nevertheless, the studies were conducted before 2008 and are limited by relatively small sample sizes (*n* = 90–349). There is also a lack of studies assessing the construct validity and responsiveness of the EQ-5D-3L descriptive system. Consequently, this study aimed to add to the evidence base regarding the measurement properties of the EQ-5D-3L (index and descriptive system), as well as EQ VAS, by presenting updated results for construct validity and responsiveness in a real-world large population of patients with AS.

## Methods

### Study design

This study is based on real-world register data from the Swedish Rheumatology Quality Register (SRQ) [22]. It follows COSMIN’s guidelines for evaluating the construct validity and responsiveness of patient-reported outcome measures (PROMs) [17, 23–25]. Construct validity was assessed by analysing convergent validity and known-groups validity. Convergent validity refers to the extent to which EQ-5D-3L and EQ VAS correlate with the comparator instruments [17]. Known-groups validity refers to EQ-5D-3L and EQ VAS ability to find expected differences between groups known to differ [17]. The analyses of construct validity and responsiveness were based on predetermined hypotheses regarding the expected results of the EQ-5D-3L and comparator instruments measuring *similar* or *related* constructs: disease activity (measured with Bath Ankylosing Spondylitis Disease Activity Index (BASDAI) and Ankylosing Spondylitis Disease Activity Score (ASDAS)), physical function (measured with BASFI), and pain, fatigue, and general health (measured with visual analogue scales, VAS). Drawing on theoretical frameworks, existing literature, and input from clinical and methodological experts, as well as patient research partners, predetermined hypotheses were developed. Responsiveness was also assessed by calculating the area under the receiver operating characteristic curve (AUC). At least 75% of the hypotheses had to be confirmed to support construct validity and responsiveness [17, 24]. A study protocol describing the hypotheses and analysis plan has been published at ClinicalTrials.gov, ClinicalTrials.gov ID NCT06568029. Ethical approval for the study was granted by the Swedish Ethical Review Authority (registration number: 2023-04394-01). As the study is based on registry data, no informed consent was needed for this study.

### Study population and data

Data were extracted from the SRQ and included information from healthcare visits where the patient was 18 years or older, was diagnosed with AS, and had at least one complete measurement of EQ-5D-3L and BASDAI at the same visit. The data were collected through a self-service digital platform where patients self-report information related to their diagnosis in conjunction with their visit, and the healthcare provider supplements with clinical information regarding the patient’s health. For construct validity analyses, the latest available complete record was chosen when more than one existed. Responsiveness analyses were based on the first two complete records within the initial year after diagnosis, a period in which health changes are expected. The SRQ inclusion date served as a substitute in cases when the diagnosis date was not recorded.

### Outcome measurement instruments

This study included the following instruments: EQ-5D-3L, BASDAI and ASDAS CRP (both measure disease activity), BASFI (physical function), and VAS for pain, fatigue, and general health.

#### EQ-5D-3L

The first part of the EQ-5D-3L is a descriptive system comprising five dimensions of health: mobility, self-care, usual activities, pain/discomfort, and anxiety/depression [6, 7]. Each one of the five dimensions is represented by an item with three response levels: no problem (level 1), some problems (level 2), or extreme problems (level 3). The answers to the five items can be combined into a 5-digit health state profile (e.g. 11222) and the combinations can produce 243 unique health profiles. With the use of a value set, the 5-digit health profile can be transformed into an index value. An index value of 1 represents full health, and 0 represents a value considered to be of equal value to being dead. In this study, the EQ-5D-3L UK value set was used for the main analyses [26]. This is the value set for EQ-5D-3L recommended by the Swedish HTA agency, The Dental and Pharmaceutical Benefits Agency (TLV) [27–29]. The second part of the EQ-5D-3L consists of the EQ VAS, in which the respondent records their self-rated health on a VAS between 0 (“the worst health you can imagine”) and 100 (“the best health you can imagine”) [6, 7]. In this study, patients completed the Swedish version of the EQ-5D-3L.

#### Disease activity

BASDAI is developed to measure patient-reported disease activity in patients with AS and includes questions about fatigue, pain, swelling, and stiffness [30, 31]. ASDAS, recommended by the Core Outcome Set (COS) for axSpA [32], is a composite instrument developed to measure disease activity in patients with AS and contains patient-reported information about pain, swelling, stiffness, and disease activity, as well as laboratory test results about inflammation [33]. Support for reliability, construct validity, and responsiveness of BASDAI and ASDAS has been found in several international studies [30, 31, 33–43]. A Swedish study also found support for reliability, construct validity, and responsiveness of BASDAI [44]. BASDAI ranges from 0 to 10, and ASDAS typically ranges from 0 to around 7 [45]. For both instruments, a higher value indicates higher disease activity.

#### Physical function

BASFI is a PROM, measuring physical function in terms of mobility, usual activities, and self-care [46], and is recommended by the COS for axSpA [32]. Support for reliability, construct validity, and responsiveness of BASFI in patients with AS has been found in several international studies and a Swedish study [37, 42, 43, 46–48]. BASFI ranges from 0 to 10, and a higher value indicates more problems with physical function.

#### Pain, fatigue, and general health

The SRQ also collects data on three patient-reported VAS for pain, fatigue, and general health over the past week, scored from 0 (no problem) to 100 (worst imaginable), see supplementary material for the three VAS [49]. Although there is a lack of studies assessing the construct validity and responsiveness of these VAS in AS, these were included due to the relevance of these symptoms to the patient population.

### Analyses

Analyses were performed on available cases. Data were analysed with IBM SPSS Statistics, Version 29.

#### Convergent validity

Convergent validity was assessed by testing predetermined hypotheses regarding the expected direction and strength of the correlation of the results from the EQ-5D-3L (index and descriptive system) and EQ VAS with the results from comparator instruments measuring similar or related constructs at the same health care visit [17, 23, 25], see Table 1. At least moderate correlations (≥ 0.3^1^) were expected for *related* constructs, and strong correlations (≥ 0.5^**1**^) for *similar* constructs [25]. Spearman’s Rho (r) was used for the correlations between the EQ-5D-3L index and EQ VAS and the comparator instruments, and Kendall’s Tau (τ) was used for correlations between the dimensions in the descriptive system and the comparator instruments.

**Table 1:** Predetermined hypotheses for construct validity and responsiveness of the EQ-5D-3L and the EQ VAS

Comparator instruments	EQ VAS	EQ-5D-3L descriptive system					EQ-5D-3L index
		Mobility	Self-care	Usual activities	Pain/ discomfort	Anxiety/ depression	
BASDAI	≤-0.3	≥0.3	≥0.3	≥0.3	≥0.3		≤-0.3
ASDAS CRP	≤-0.3	≥0.3	≥0.3	≥0.3	≥0.3		≤-0.3
BASFI	≤-0.3	≥0.3	≥0.3	≥0.3	≥0.3		≤-0.3
VAS pain	≤-0.3	≥0.3	≥0.3	≥0.3	≥0.5		≤-0.3
VAS fatigue	≤-0.3	≥0.3	≥0.3	≥0.3		≥0.3	≤-0.3
VAS general health	≤-0.5						≤-0.5

ASDAS CRP, Ankylosing Spondylitis Disease Activity Score CRP; BASDAI, Bath Ankylosing Spondylitis Disease Activity Index; BASFI, Bath Ankylosing Spondylitis Functional Index; VAS, Visual Analogue Scale

#### Known-groups validity

To assess known-groups validity, scores from the EQ-5D-3L were compared across subgroups of patients defined by their disease activity or physical functioning. Three groups were created based on ASDAS CRP, BASDAI and BASFI. It was hypothesised that individuals with lower disease activity or better physical function (< 2.1 ASDAS CRP, < 4 BASDAI or < 4 BASFI) would report fewer problems in the dimensions mobility, self-care, usual activities, and pain/discomfort, as well as score higher on both the EQ-5D-3L index and the EQ VAS, than patients with higher disease activity or lower physical function (≥ 2.1 ASDAS CRP, ≥ 4 BASDAI or ≥ 4 BASFI).

Furthermore, it was hypothesised that the difference in EQ-5D-3L index or EQ VAS between the groups would be at least as large as minimal important differences (MIDs). MIDs reflect the smallest difference in score that patients perceive as important [50]. Specifically, a difference of ≥ 0.05 in the EQ-5D-3L index and ≥ 10.3 points in the EQ VAS was considered indicative of known-groups validity. The MID for the EQ-5D-3L index was based on patients with rheumatoid arthritis (RA), while the MID for the EQ VAS was based on MIDs reported in musculoskeletal disorder populations [51, 52]. To examine differences in the distribution of individual-level ordinal responses within the EQ-5D-3L descriptive system, the Mann–Whitney U test was applied. This non-parametric approach was chosen to account for the ordinal data [53]. Effect sizes (r) were calculated to quantify the magnitude of differences in response distribution between the groups, with thresholds of ≥ 0.1, ≥ 0.3, and ≥ 0.5 representing small, medium, and large effects, respectively [54, 55]. To support the hypotheses regarding the dimensions, at least a small effect size was required. 

#### Responsiveness

To assess responsiveness, we examined how changes over time in EQ-5D-3L and EQ VAS scores correlated with corresponding changes in the scores from comparator instruments, applying the same hypotheses used for convergent validity (Table 1) [17, 23, 25]. The ability of the EQ-5D-3L and the EQ VAS to discriminate between improved and non-improved patients, defined by changes in disease activity or physical function, was assessed via AUC, with predetermined hypotheses of an AUC ≥ 0.70 for BASDAI, ASDAS CRP, and BASFI [17, 23, 25]. The criteria for improvement for BASDAI and BASFI were > 50% or more than two points improvement on the scale, and improvement for ASDAS CRP was ≥ 1.1 [56].

#### Sensitivity analyses

The Swedish experience-based value set for the EQ-5D-3L index [57] was used in a sensitivity analysis. For the assessment of responsiveness, an additional analysis was performed, including only patients with a documented diagnosis date, thereby confirming that the time from diagnosis was no longer than 365 days.

## Results

### Construct validity of the EQ-5D-3L index and the descriptive system

**Fig. 1:**
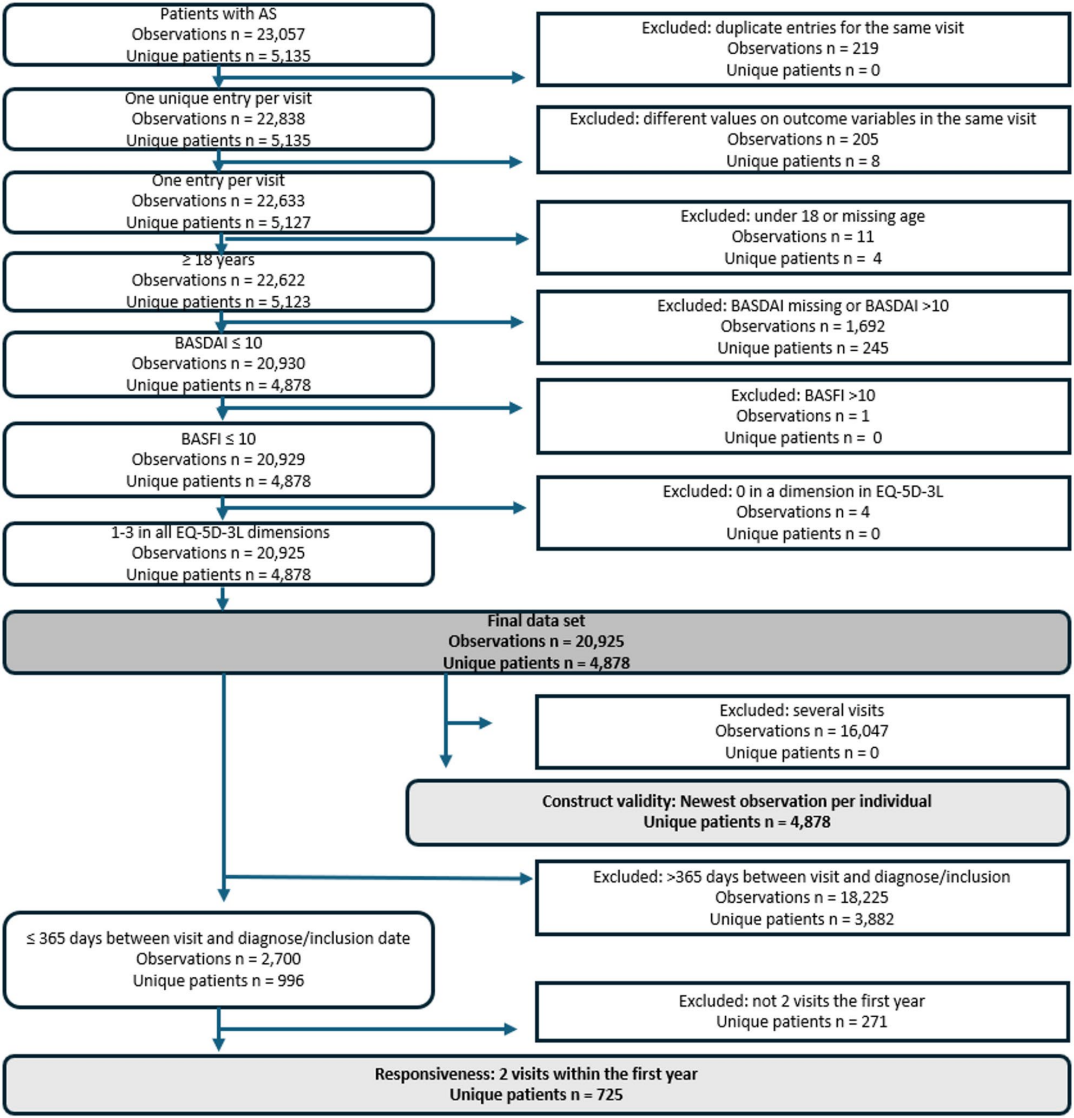
Flowchart describing the study population. AS, Ankylosing Spondylitis; BASDAI, Bath Ankylosing Spondylitis Disease Activity Index; BASFI, Bath Ankylosing Spondylitis Functional Index

The data for the analysis of construct validity of EQ-5D-3L included the last complete measurement of 4,878 patients (Fig. 1). The mean age was 49.2 years (SD 14.5), and 32.7% were female (Table 2). The mean EQ-5D-3L index at the last visit was 0.64 (SD 0.31). Pain/discomfort was the most reported problem (81.9%), followed by anxiety/depression (48.2%). At the last complete visit, 13.8% of patients reported full health (Table 2).

**Table 2:** Background characteristics

	Population for responsiveness (newly diagnosed)		Population for construct validity *n* = 4,878
	Visit 1 *n* = 725	Visit 2 *n* = 725	
Female (%)	36.0		32.7
Age at diagnosis, mean (SD)	37.02 (12.69) ^a^		35.06 (12.17) ^a^
Age at EQ-5D-3L registration, mean (SD)	39.63 (13.72)	40.02 (13.70)	49.23 (14.45)
Days between visit 1 and 2 (SD)	141.82 (73.63)		
BASDAI, mean (SD)	4.60 (2.30)	3.60 (2.47)	3.57 (2.40)
ASDAS CRP, mean (SD)	2.83 (1.08)	2.18 (1.10)	2.15 (1.08)
BASFI, mean (SD)	3.42 (2.50)	2.65 (2.47)	2.72 (2.55)
Pain, mean (SD)	50.03 (25.64)	36.55 (27.64)	35.84 (27.04)
Fatigue, mean (SD)	53.08 (27.67)	41.89 (28.98)	40.40 (29.17)
General health, mean (SD)	48.48 (25.03)	36.84 (27.31)	36.74 (26.85)
EQ-5D-3L Index, mean (SD)	0.510 (0.340)	0.619 (0.313)	0.642 (0.305)
EQ VAS, mean (SD)	59.84 (21.05)	66.34 (19.78)	64.49 (22.56)
EQ-5D-3L descriptive system			
Mobility			
No problem	45.7%	62.1%	64.2%
Some problems	54.1%	37.7%	35.5%
Unable	0.3%	0.3%	0.2%
Self-care			
No problem	79.6%	84.1%	86.5%
Some problems	19.9%	14.8%	12.6%
Unable	0.6%	1.1%	0.9%
Usual activities			
No problem	54.6%	64.3%	68.5%
Some problems	39.0%	31.9%	28.0%
Unable	6.3%	3.9%	3.5%
Pain/discomfort			
No problems	7.7%	16.7%	18.1%
Moderate problems	65.5%	68.3%	67.4%
Extreme problems	26.8%	15.0%	14.5%
Anxiety/depression			
No problems	37.9%	47.6%	51.8%
Moderate problems	52.0%	43.9%	41.8%
Extreme problems	10.1%	8.6%	6.4%
Full health	5.2%	12.0%	13.8%
Year of the visit	Number of patients having a visit each year		
2008	1	0	0
2009	27	14	8
2010	61	44	31
2011	31	52	38
2012	37	30	71
2013	54	44	103
2014	81	78	124
2015	60	64	157
2016	59	63	191
2017	71	66	243
2018	60	60	336
2019	42	50	441
2020	29	37	280
2021	45	33	416
2022	24	37	668
2023	32	27	923
2024	11	26	848

ASDAS CRP, Ankylosing Spondylitis Disease Activity Score CRP; BASDAI, Bath Ankylosing Spondylitis Disease Activity Index; BASFI, Bath Ankylosing Spondylitis Functional Index; SD, Standard deviation

^a^ 40% had missing date of diagnosis

#### Convergent validity

The EQ-5D-3L index demonstrated strong correlations (≥ 0.5) with all comparator instruments (BASDAI, ASDAS CRP, BASFI, and VAS pain, fatigue, and general health), in line with the hypothesised strength (Table 3). Of the 20 hypothesised correlations between responses to the items in the descriptive system of EQ-5D-3L and comparator instruments, 18 were moderate or strong (as hypothesised). Only the self-care dimension showed weaker-than-expected correlations with VAS pain (τ = 0.28) and VAS fatigue (τ = 0.27).

**Table 3:** Correlations between the EQ-5D-3L, the EQ VAS, and the comparator instruments for convergent validity

Comparator instruments	EQ VAS *n* = 2,651^a^	EQ-5D-3L descriptive system					EQ-5D-3L Index *n* = 4,878
		Mobility *n* = 4,878	Self-care *n* = 4,878	Usual activities *n* = 4,878	Pain/ discomfort *n* = 4,878	Anxiety/ depression *n* = 4,878	
BASDAI *n* = 4,878	**-0.63**	**0.45**	**0.31**	**0.44**	**0.55**	(0.38)	**-0.73**
ASDAS CRP *n* = 3,975	**-0.60**	**0.45**	**0.31**	**0.42**	**0.51**	(0.33)	**-0.69**
BASFI *n* = 4,815	**-0.61**	**0.54**	**0.38**	**0.49**	**0.48**	(0.30)	**-0.71**
VAS pain *n* = 4,692	**-0.57**	**0.42**	0.28	**0.40**	**0.56**	(0.32)	**-0.69**
VAS fatigue *n* = 4,622	**-0.59**	**0.38**	0.27	**0.41**	(0.47)	**0.37**	**-0.66**
VAS general health *n* = 4,691	**-0.63**	(0.42)	(0.30)	(0.43)	(0.52)	(0.38)	**-0.71**

Bold font indicates that the hypothesis was supported. Regular font indicates that the hypothesis was not supported. Correlations in brackets indicate that no hypothesis was made. Spearman’s Rho was used for the EQ-5D-3L index value and the EQ VAS, and Kendall’s Tau was used for the descriptive system. All correlations are significant at the 0.01 level (2-tailed). ASDAS CRP, Ankylosing Spondylitis Disease Activity Score CRP; BASDAI, Bath Ankylosing Spondylitis Disease Activity Index; BASFI, Bath Ankylosing Spondylitis Functional Index; VAS, Visual Analogue Scale. ^a^ The number of observations for the EQ VAS is lower due to later start of data collection

#### Known-groups validity

The mean EQ-5D-3L index was higher in patients with lower disease activity or higher physical function compared to those with higher disease activity or lower physical function. The differences between the groups were 0.36 based on BASDAI, 0.33 based on ASDAS CRP, and 0.39 based on BASFI, which means that they significantly exceeded the MID of 0.05 (Table 4). In all five dimensions, patients with higher disease activity or lower physical function also reported more problems. The differences in reported problems were statistically significant, and all effect sizes were, as hypothesised, at least small (≥ 0.1). The smallest effect sizes were observed in the dimensions self-care (*r* = 0.31, ASDAS CRP) and anxiety/depression (*r* = 0.31, BASFI), while the largest effect size was observed in mobility (*r* = 0.6, BASFI).

In total, 39 of the 41 (95%) hypotheses for construct validity were supported for the EQ-5D-3L.

**Table 4:** Mean EQ-5D-3L index and EQ VAS, and responses in the descriptive system of the EQ-5D-3L (known-groups validity)

		BASDAI			ASDAS CRP			BASFI		
		< 4 *n* = 2,906	≥ 4 *n* = 1,972		< 2.1 *n* = 2,081	≥ 2.1 *n* = 1,894		< 4 *n* = 3,465	≥ 4 *n* = 1,350	
	**Mean EQ-5D-3L index (SD)**	0.786 (0.164)	0.429 (0.337)	*p* < 0.001 1.44^a^ **0.357** ^**b**^	0.797 (0.163)	0.471 (0.332)	*p* < 0.001 1.27^a^ **0.326** ^**b**^	0.753 (0.202)	0.363 (0.336)	*p* < 0.001 1.58^a^ **0.390** ^**b**^
Mobility	No problem (%)	83.1	36.4	*p* < 0.001 z = 33.4 ***r*** **= 0.48**	86.1	39.9	*p* < 0.001 z = 30.4 ***r*** **= 0.48**	82.4	18.7	*p* < 0.001 z = 41.5 ***r*** **= 0.60**
	Some problems (%)	16.9	63.1		13.8	59.6		17.5	80.7	
	Unable (%)	0.1	0.5		0.0	0.5		0.1	0.7	
Self-care	No problem (%)	95.9	72.7	*p* < 0.001 z = 23.2 ***r*** **= 0.33**	96.4	75.0	*p* < 0.001 z = 19.5 ***r*** **= 0.31**	97.0	60.4	*p* < 0.001 z = 33.5 ***r*** **= 0.48**
	Some problems (%)	3.5	26.1		2.8	23.8		2.5	37.9	
	Unable (%)	0.6	1.3		0.8	1.2		0.5	1.7	
Usual activities	No problem (%)	86.7	41.6	*p* < 0.001 z = 33.4 ***r*** **= 0.48**	88.5	46.7	*p* < 0.001 z = 28.4 ***r*** **= 0.45**	84.3	28.5	*p* < 0.001 z = 38.0 ***r*** **= 0.55**
	Some problems (%)	12.6	50.8		10.9	46.3		15.0	60.9	
	Unable (%)	0.7	7.6		0.6	7.0		0.7	10.6	
Pain/ discomfort	No problem (%)	28.5	2.8	*p* < 0.001 z = 35.1 ***r*** **= 0.50**	31.6	3.3	*p* < 0.001 z = 31.4 ***r*** **= 0.50**	23.9	3.3	*p* < 0.001 z = 30.4 ***r*** **= 0.44**
	Moderate problems (%)	70.4	63.1		67.6	67.1		71.7	57.0	
	Extreme problems (%)	1.2	34.1		0.9	29.6		4.4	39.7	
Anxiety/ depression	No problem (%)	67.2	29.0	*p* < 0.001 z = 27.4 *r* = 0.39	68.1	34.3	*p* < 0.001 z = 22.0 *r* = 0.35	60.9	28.5	*p* < 0.001 z = 21.7 *r* = 0.31
	Moderate problems (%)	30.8	58.0		29.7	54.9		35.9	57.1	
	Extreme problems (%)	2.0	13.0		2.2	10.8		3.2	14.4	
		*n* = 1,598	*n* = 1,053		*n* = 1,183	*n* = 961		*n* = 1,976	*n* = 674	
	**EQ VAS**^**c**^ **(SD)**	73.89 (18.41)	50.22 (20.71)	*p* < 0.001 1.22^a^ **23.67** ^**b**^	74.69 (18.36)	52.05 (21.28)	*p* < 0.001 1.15^a^ **22.64** ^**b**^	70.77 (19.34)	46.13 (21.25)	*p* < 0.001 1.24^a^ **24.64** ^**b**^

Bolded effect size or difference in the EQ-5D-3L index or the EQ VAS indicates that the hypothesis was supported. No hypotheses were made for the anxiety/depression dimension. Proportions of respondents reporting problems in the dimensions were presented descriptively to aid interpretation but were not used as the basis for statistical testing. ASDAS CRP, Ankylosing Spondylitis Disease Activity Score CRP; BASDAI, Bath Ankylosing Spondylitis Disease Activity Index; BASFI, Bath Ankylosing Spondylitis Functional Index; p, p-value; r, effect size; SD, standard deviation; VAS, Visual Analogue Scale; Z, Z-score Mann-Whitney U test. ^a^ Effect size was calculated using Cohen’s d, ^b^ Difference in mean EQ-5D-3L index /EQ VAS between the groups, ^c^ The number of observations for the EQ VAS is lower due to later start of data collection

### Responsiveness of the EQ-5D-3L index and the descriptive system

A total of 725 patients had two complete visits within 365 days of diagnosis, with a mean interval of 142 days (SD 73.6). From the first to the second visit, the mean EQ-5D-3L index increased from 0.51 (SD 0.34) to 0.62 (SD 0.31). Despite improvements, many patients still reported problems with pain/discomfort (from 92.3% to 83.3%) and anxiety/depression (from 62.1% to 52.5%) at the second visit. The proportions of patients who at the first visit reported no problem with mobility, self-care, usual activities, and anxiety/depression were 45.7%, 79.6%, 54.6% and 37.9% respectively, indicating ceiling effects (Table 2). The proportion reporting full health increased from 5.2% to 12.0% between the two visits.

All correlations between the changes in the EQ-5D-3L index between the two visits and the corresponding changes in the comparator instruments were strong and in line with the hypothesised strength, except for the moderate correlation with VAS fatigue (*r* = -0.47) (Table 5). All correlations between the changes over time in the EQ-5D-3L dimension self-care and the comparator instruments were below the expected correlation of at least 0.3 (ranging from τ = 0.11 to 0.20). Generally, changes in the EQ-5D-3L dimension pain/discomfort and in the comparator instruments showed the highest correlations. However, it only showed a moderate correlation (τ = 0.39) with changes in VAS pain, which was lower than the hypothesised strong correlation (≥ 0.5).

Figure S1 in the supplementary material presents Sankey diagrams showing how patient responses in the EQ-5D-3L descriptive system changed among those who improved and those who did not improve according to BASDAI.

**Table 5:** Correlations between changes in the EQ-5D-3L, the EQ VAS, and changes in the comparator instruments for responsiveness

Comparator instruments	EQ VAS^a^ *n* = 93	EQ-5D-3L descriptive system					EQ-5D-3L Index *n* = 725
		Mobility *n* = 725	Self-care *n* = 725	Usual activities *n* = 725	Pain/ discomfort *n* = 725	Anxiety/ Depression *n* = 725	
BASDAI *n* = 725	**-0.43**	**0.32**	0.17	0.27	**0.38**	(0.29)	**-0.57**
ASDAS CRP *n* = 512	**-0.54**	**0.31**	0.18	0.26	**0.37**	(0.26)	**-0.56**
BASFI *n* = 667	**-0.37**	**0.36**	0.20	**0.31**	**0.36**	(0.24)	**-0.55**
VAS pain *n* = 677	**-0.60**	**0.31**	0.13	0.27	0.39	(0.24)	**-0.53**
VAS fatigue *n* = 555	**-0.41**	0.24	0.11	0.24	(0.29)	0.28	**-0.47**
VAS general health *n* = 678	**-0.58**	(0.31)	(0.13)	(0.25)	(0.36)	(0.23)	**-0.51**

Bold font indicates that the hypothesis was supported. Regular font indicates that the hypothesis was not supported. Correlations in brackets indicate that no hypothesis was made. Spearman’s Rho was used for the EQ-5D-3L index and the EQ VAS, and Kendall’s Tau was used for the descriptive system. All correlations are significant at the 0.01 level (2-tailed)

ASDAS CRP, Ankylosing Spondylitis Disease Activity Score CRP; BASDAI, Bath Ankylosing Spondylitis Disease Activity Index; BASFI, Bath Ankylosing Spondylitis Functional Index; VAS, Visual Analogue Scale. ^a^ The number of observations for the EQ VAS is lower due to later start of data collection

In the analysis for the AUC, 268 patients (37.0%) were classified as improved according to BASDAI, 163 (31.9%) according to ASDAS CRP, and 259 (38.8%) according to BASFI. The AUC was above 0.7 in all three analyses (Table 6). This indicates that the EQ-5D-3L index could differentiate between patients who improved and those who did not improve (defined based on the measurements from BASDAI, ASDAS CRP, and BASFI). In total, 17 of the 29 hypotheses (59%) for responsiveness were supported for the EQ-5D-3L.

**Table 6:** Area under the receiver operating characteristic curve for the EQ-5D-3L index

	AUC (95% CI)
BASDAI *n* = 725	**0.79 (0.75–0.82)**
ASDAS CRP *n* = 511	**0.76 (0.71–0.80)**
BASFI *n* = 667	**0.75 (0.71–0.78)**

Bold font indicates that the hypothesis was supported

ASDAS CRP, Ankylosing Spondylitis Disease Activity Score CRP; AUC, Area Under the Curve; BASDAI, Bath Ankylosing Spondylitis Disease Activity Index; BASFI, Bath Ankylosing Spondylitis Functional Index; CI, Confidence Interval

### Sensitivity analyses of the EQ-5D-3L index and the descriptive system

Calculating the EQ-5D-3L index with the Swedish experience-based value set produced results consistent with the main analysis (see Table S1-S3 in supplementary material). Similarly, restricting the responsiveness analysis to patients with a recorded diagnosis date yielded comparable findings (see Table S4 and S5 in supplementary material).

### Construct validity of the EQ VAS

The data for the analysis of construct validity of the EQ VAS consisted of the last complete measurement of 2,651 patients. The mean EQ VAS at the last complete measurement was 64.5 (SD 22.6) (Table 2). The EQ VAS demonstrated strong correlations (≥ 0.5) with all comparator instruments (BASDAI, ASDAS CRP, BASFI, and VAS pain, fatigue, and general health), in line with the hypothesised strength (Table 3). The differences in mean EQ VAS between patients with high versus low disease activity or low versus high physical function measured with BASDAI, ASDAS CRP, and BASFI were 23.7, 22.7, and 24.6, respectively, exceeding the MID value of 10.3 (Table 4). All nine hypotheses were supported by the data.

### Responsiveness of the EQ VAS

Responsiveness of the EQ VAS was assessed based on measures from 93 patients. The mean EQ VAS increased from 59.8 (SD 21.1) on the first visit to 66.3 (SD 19.8) on the second visit (Table 2). Correlations between changes in the EQ VAS and all the comparator instruments were at least moderate (Table 5). The AUC for EQ VAS could not be calculated due to the limited sample size. All six hypotheses for responsiveness were supported by the data.

### Sensitivity analyses of the EQ VAS

A sensitivity analysis including only patients with a recorded diagnosis date confirmed the main findings (see Table S4 in supplementary material).

## Discussion

The findings of this registry-based study, using data from routine healthcare for patients with AS, contribute to the evidence base on the measurement properties of EQ-5D-3L and EQ VAS. In line with previous research, our findings support the construct validity of the EQ-5D-3L index and the EQ VAS [19–21]. Although the sample size was small, our findings also confirmed previous studies supporting the responsiveness of the EQ VAS in this patient group [19, 20]. To our knowledge, this is the first study to evaluate the construct validity and responsiveness of the descriptive system of EQ-5D-3L in this patient population following the guidelines by COSMIN [17, 23, 25]. Given that the results from the EQ-5D-3L descriptive system are recommended for use in clinical settings and are used in research and reports from national quality registries [12, 13, 15], it is important to understand how well the dimensions can detect differences between groups and over time. Furthermore, the EQ-5D-3L index, which is frequently used in clinical studies and economic evaluations, is derived from the responses to the items in all five dimensions in the descriptive system. Thus, evidence of the construct validity and responsiveness of the dimensions in the descriptive system is also important to ensure that the index can capture changes in the patient groups for which it is used. Importantly, we found support for construct validity of the EQ-5D-3L descriptive system, as the dimension-level results correlated as expected with the results of the comparator instruments and distinguished between patients with different levels of disease activity or physical function. Nevertheless, the findings regarding the responsiveness of the dimensions self-care and usual activities raise concern regarding the descriptive system as well as the index. Unlike Haywood et al. [20], we found that the EQ-5D-3L index could detect changes over time, as all hypotheses (9 of 9) related to the EQ-5D-3L index were supported. However, several hypotheses related to the EQ-5D-3L dimensions, especially self-care and usual activities, were not supported. Overall, only 17 of the 29 (59%) hypotheses related to the EQ-5D-3L responsiveness were supported. Similar results regarding the responsiveness of the descriptive system were found in a study using the same method and SRQ data, but among patients with PsA (Degerlund-Maldi, unpublished data, 2025). In the assessment for construct validity, the differences in EQ-5D-3L between disease activity and physical function groups were larger than expected, and several correlations were strong. For example, the EQ-5D-3L dimension pain/discomfort demonstrated strong correlations with both disease activity instruments, BASDAI and ASDAS CRP. These findings are consistent with the content of the respective instruments, as both BASDAI and ASDAS include items related to pain and discomfort [30, 31, 33]. In addition, we found moderate correlations between the dimension anxiety/depression and all comparator instruments. Furthermore, we observed differences in reported problems with anxiety/depression between the groups based on disease activity or physical function. Some correlations were weaker than expected. First, the EQ-5D-3L dimension self-care demonstrated weaker correlations than hypothesised in the analysis of convergent validity and responsiveness. In the analysis of convergent validity, self-care showed weak correlations with VAS pain and VAS fatigue. In the responsiveness analysis, correlations between self-care and all comparator instruments were lower than expected. While limitations in VAS pain and VAS fatigue could be a factor, prior research in related populations (e.g., RA) supports the construct validity and responsiveness of these VAS [58–66]. Our hypotheses were based on the assumption that pain and fatigue in AS would impair a person’s ability to perform self-care activities. This assumption is supported by previous literature indicating that pain and fatigue can affect patients’ ability to perform self-care [67]. Another possible explanation for the lack of responsiveness is the substantial ceiling effect observed in this dimension. At the first visit, almost 80% reported no problems in the EQ-5D-3L item self-care, meaning that many patients could not report improvement in this item despite reporting improvements in the comparator instruments. Large ceiling effects in the descriptive system and, specifically, the self-care item, have been observed in patients with AS [19] and in other populations [68, 69], and to address the lack of responsiveness, a new version, the EQ-5D-5L, with five response options, has been developed [70]. In addition, there is ongoing work developing additional dimensions that can be added to the five dimensions in EQ-5D, known as EQ-5D bolt-ons [71]. The purpose of the bolt-ons is to improve the measurement properties of the EQ-5D in certain populations, while keeping the instrument brief. For example, given that fatigue has been reported as an important symptom of AS, the development of a bolt-on for tiredness could be relevant for this patient group [72]. The low psychometric performance of the self-care item could also be attributed to the phrasing of the item in the Swedish version, which is slightly different from the English one. Second, the EQ-5D-3L dimension for usual activities exhibited consistently weaker correlations for all but one of the hypotheses for the analysis of responsiveness (*τ* = 0.31 BASFI). Lastly, despite hypothesising a strong correlation, the pain/discomfort dimension demonstrated only a moderate correlation (*τ *= 0.39) with VAS pain, indicating potential limitations in its ability to detect changes in pain.

A strength of our study is that we followed the guidelines from COSMIN, which ensures a rigorous and standardised methodological approach [17, 23, 25]. We used well-known comparator instruments such as ASDAS, BASDAI, and BASFI, which have demonstrated construct validity and responsiveness, for our analyses. The hypotheses were developed in collaboration with clinical and methodological experts as well as patient research partners, which strengthens their quality. Another strength is the use of both the UK [26] and the Swedish value set [57] for estimating the EQ-5D-3L index. The sensitivity analysis showed that the results are robust, regardless of which value set is used. Lastly, this study was based on data from a large national sample collected within routine clinical care, including patients between the years 2008–2024. This increases the generalisability of the results and reduces the risk of selection bias.

The study also has some limitations. The responsiveness analysis included AS patients diagnosed within a year before their first and second visits. However, patients lacking a date of diagnosis were selected based on their date of inclusion in the SRQ. These patients may have had their diagnosis for longer than a year. However, a proportion of the patients had an improvement according to the comparator instruments, and a sensitivity analysis only including patients with a date of diagnosis showed consistent results. Furthermore, in the analysis of known-groups validity and responsiveness, we grouped patients based on information regarding disease activity or physical function. The patients might also differ in other important ways, such as the presence of comorbidities. As data on comorbidity were not available and could not be adjusted for, scores from the EQ-5D-3L and the EQ VAS may also reflect other health conditions beyond AS-related disease activity or physical function. Therefore, we recommend that future studies adjust for comorbidities. Lastly, we employed three VAS that measured pain, fatigue, and general health. While data supporting their construct validity and responsiveness in patients with AS are lacking, such data are available for patients with RA [58–66].

These findings have important implications for selecting instruments to measure HRQoL in patients with AS and for interpreting EQ-5D-3L results in this population. The results support the use of the descriptive system, the EQ-5D-3L index, and the EQ VAS to describe and assess differences in HRQoL between patients with different disease activity and physical function. However, the findings suggest caution when interpreting changes over time using the descriptive system and EQ-5D-3L index. The lack of responsiveness in the dimensions self-care and usual activities suggests that the EQ-5D-3L index may not fully capture the effects over time of interventions affecting these dimensions. Given its expanded response levels, the 5-level version, EQ-5D-5L, has the potential to address some of the limitations observed with the EQ-5D-3L, particularly in capturing changes over time in the descriptive system. Future research should explore the construct validity and responsiveness of the EQ-5D-5L in patients with AS.

## Conclusion

Our findings support the use of the EQ-5D-3L index, descriptive system, and EQ VAS for measuring health and HRQoL cross-sectionally, as well as the use of the EQ VAS for capturing longitudinal changes. The results showed that the EQ-5D-3L index was sensitive to change, as all predetermined hypotheses for the index were supported. However, the dimensions self-care and usual activities demonstrated limited responsiveness. As the EQ-5D-3L index is calculated from all five dimensions, it may not fully reflect changes resulting from interventions that impact self-care and usual activities.

## Supplementary Information

Below is the link to the electronic supplementary material.

Supplementary Material 1

## Data Availability

Access to data is restricted by Swedish law. General information about obtaining access to data is available from the corresponding author.

## Footnotes

1 For negative correlations moderate correlations were defined as ≤-0.3﻿ and strong correlations as ≤-0.5.

## Abbreviations

AS: Ankylosing spondylitis
ASDAS: Ankylosing Spondylitis Disease Activity Score
AUC: Area under the curve
BASDAI: Bath Ankylosing Spondylitis Disease Activity Index
BASFI: Bath Ankylosing Spondylitis Functional Index
COS: Core Outcome Set
EQ VAS: EQ visual analogue scale
HRQoL: Health-related quality of life
MID: Minimal important difference
PROM: Patient-reported outcome measure
PsA: Psoriatic arthritis
RA: Rheumatoid arthritis
SD: Standard deviation
SRQ: Swedish Rheumatology Quality Register
VAS: Visual analogue scale
