# Supplementary material for: The value of EQ-5D-3L and EQ VAS as a patient-reported outcome measure for patients with ankylosing spondylitis in routine healthcare: an evaluation of construct validity and responsiveness based on the Swedish Rheumatology Quality Register
Source: J Patient Rep Outcomes. 2026 Feb 7;10:51. doi: 10.1186/s41687-026-01009-0 (PMC13057080; doi:10.1186/s41687-026-01009-0)
Supplement: Supplementary file 1 — Supplementary Material 1 [file 41687_2026_1009_MOESM2_ESM.docx]

Supplementary material: Visual analogue scales (VAS)

Authors' translation of the three VAS included in the study.

Question:
“How much pain have you experienced over the past week due to your rheumatic disease?”

Scale where the patient indicates their answer:
No pain --------------------------------------------------------------------------------------------- Worst pain imaginable

Question:
“How fatigued have you been over the past week due to your rheumatic disease?”

Scale where the patient indicates their answer:
No fatigue -------------------------------------------------------------------------------------- Worst fatigue imaginable

Question:
”How have you generally felt over the past week, considering your rheumatic disease?”

Scale where the patient indicates their answer:
Completely well ----------------------------------------------------------------------------------- As bad as imaginable

Supplementary material: Sensitivity analyses

Table S1. Correlations between the EQ-5D-3L index (Swedish experience-based value set) and the comparator instruments for convergent validity and responsiveness

| Convergent validity | | Responsiveness | |
| --- | --- | --- | --- |
| Comparator instruments | EQ-5D-3L Index  n = 4,878 | Comparator instruments | EQ-5D-3L Index  n = 725 |
| BASDAI  n = 4,878 | **-0.72** | BASDAI  n = 725 | **-0.56** |
| ASDAS CRP  n = 3,975 | **-0.68** | ASDAS CRP  n = 512 | **-0.55** |
| BASFI  n = 4,815 | **-0.72** | BASFI  n = 667 | **-0.56** |
| VAS pain  n = 4,692 | **-0.67** | VAS pain  n = 677 | **-0.52** |
| VAS fatigue  n = 4,622 | **-0.66** | VAS fatigue  n = 555 | **-0.47** |
| VAS general health  n = 4,691 | **-0.70** | VAS global  n = 678 | **-0.51** |

Bold font indicates that the hypothesis was supported. All correlations are significant at the 0.01 level (2-tailed).
ASDAS, Ankylosing Spondylitis Disease Activity Score; BASDAI, Bath Ankylosing Spondylitis Disease Activity Index; BASFI, Bath Ankylosing Spondylitis Functional Index; VAS, Visual Analogue Scale. All correlations are significant at the 0.001 level (2-tailed).

Table S2. Mean EQ-5D-3L index value (the Swedish experience-based value set)

|  | BASDAI | |  | ASDAS CRP | |  | BASFI | |  |
| --- | --- | --- | --- | --- | --- | --- | --- | --- | --- |
|  | < 4  n = 2,906 | ≥ 4  n = 1,972 |  | <2.1  n = 2,081 | ≥2.1  n = 1,894 |  | < 4  n = 3,465 | ≥ 4  n = 1,350 |  |
| Mean  EQ-5D-3L index | 0.895 | 0.728 | p < 0.001  ES 1.51^a^  0.167^b^ | 0.901 | 0.748 | p < 0.001  ES 1.34^a^  0.153^b^ | 0.882 | 0.691 | p < 0.001  ES 1.80^a^  0.191^b^ |

ASDAS, Ankylosing Spondylitis Disease Activity Score; BASDAI, Bath Ankylosing Spondylitis Disease Activity Index; BASFI, Bath Ankylosing Spondylitis Functional Index; ES, effect size; p, p-value. ^a^ Effect size calculated with Cohen’s d, ^b^ Difference in mean EQ-5D-3L index value between the groups

Table S3. Area under the receiver operating characteristic curve of the EQ-5D-3L index (the Swedish experience-based value set)

|  | AUC (95% CI) |
| --- | --- |
| BASDAI  n = 725 | **0.78 (0.74-0.81)** |
| ASDAS CRP  n = 511 | **0.75 (0.71-0.80)** |
| BASFI  n = 667 | **0.74 (0.70-0.78)** |

Bold font indicates that the hypothesis was supported. ASDAS, Ankylosing Spondylitis Disease Activity Score; BASDAI, Bath Ankylosing Spondylitis Disease Activity Index; BASFI, Bath Ankylosing Spondylitis Functional Index; CI, Confidence interval

Table S4. Correlations between changes in EQ-5D-3L and the comparator instruments including only patients with a date of diagnosis.

|  |  | EQ-5D-3L descriptive system | | | | |  |
| --- | --- | --- | --- | --- | --- | --- | --- |
| Comparator instruments | EQ VAS  n = 83^a^ | Mobility  n = 433 | Self-care  n = 433 | Usual activities  n = 433 | Pain/  discomfort  n = 433 | Anxiety/  Depression n = 433 | EQ-5D-3L Index  n = 433 |
| BASDAI  n = 433 | **-0.44** | **0.37** | 0.19 | 0.28 | **0.40** | (0.32) | **-0.62** |
| ASDAS CRP  n = 284 | **-0.60** | **0.36** | 0.21 | 0.27 | **0.39** | (0.29) | **-0.59** |
| BASFI  n = 422 | **-0.37** | **0.39** | 0.20 | **0.31** | **0.36** | (0.29) | **-0.59** |
| VAS pain  n = 410 | **-0.58** | **0.36** | 0.14 | 0.28 | 0.38 | (0.25) | **-0.53** |
| VAS fatigue  n = 392 | **-0.40** | 0.28 | 0.12 | 0.25 | (0.29) | 0.29 | **-0.49** |
| VAS general health  n = 410 | **-0.55** | (0.35) | (0.17) | (0.26) | (0.37) | (0.23) | **-0.55** |

Bold font indicates that the hypothesis was supported. Regular font indicates that the hypothesis was not supported. Correlations in brackets indicate that no hypothesis was made. Spearman’s Rho was used for the EQ-5D-3L index values and the EQ VAS, and Kendall’s Tau for the descriptive system. All correlations are significant at the 0.01 level (2-tailed). ASDAS CRP, Ankylosing Spondylitis Disease Activity Score CRP; BASDAI, Bath Ankylosing Spondylitis Disease Activity Index; BASFI, Bath Ankylosing Spondylitis Functional Index; VAS, Visual Analogue Scale. ^a^ The number of observations for the EQ VAS is lower due to later start of data collection.

Table S5. Area under the receiver operating characteristic curve of EQ-5D-3L index including only patients with a date of diagnosis.

|  | AUC (95% CI) |
| --- | --- |
| BASDAI  n = 433 | **0.82 (0.77-0.86)** |
| ASDAS CRP  n = 284 | **0.75 (0.71-0.82)** |
| BASFI  n = 422 | **0.76 (0.71-0.80)** |

Bold font indicates that the hypothesis was supported.
ASDAS, Ankylosing Spondylitis Disease Activity Score; BASDAI, Bath Ankylosing Spondylitis Disease Activity Index; BASFI, Bath Ankylosing Spondylitis Functional Index; CI, Confidence Interval


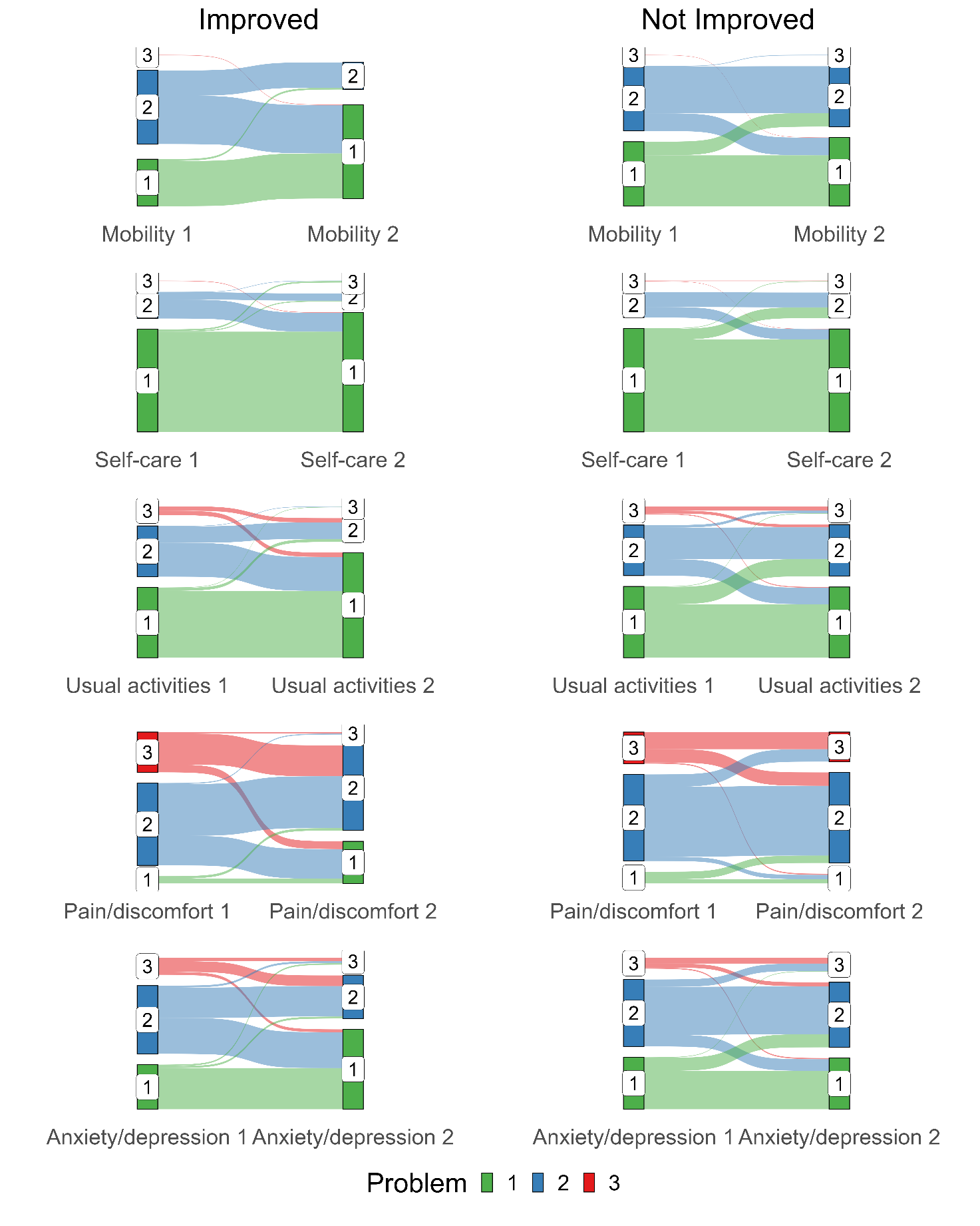


Figure S1. Sankey diagrams illustrating the transition of patient responses across EQ-5D-3L items between the first and second visit, stratified by improvement in BASDAI. Each pair of plots compares transitions for patients who improved in BASDAI (left column) versus those who did not (right column). 1 = no problem, 2 = some problems, and 3 = extreme problems.
